# Preface to the Third Voulme of the Medico-Chirurgical Review. (Analytical Series.)

**Published:** 1822-06-01

**Authors:** 


### PREFACE

TO THE

### THIRD VOLUME

OF

### THE MEDICO-CHIRURGICAL REVIEW.

### (Analytical Series.)

*This Journal has now completed the fifth year, or first lustrum of its literary existence; and it cannot look back on so eventful a period of its life without strong emotions, whether reflective on the present, or reminiscent of the past. At the commencement of the epoch alluded to, the Editor may be said to have staked his* ALL *upon the issue of the undertaking. With success in it, there* might be *success in other things—with its failure, ruin* must have *ensued —not only to himself, but to a large family! There are probably but few, whose organization of nerves would enable them to contemplate such a posture of their own affairs with perfect indifference,—especially when the whole history of* PERIODICAL MEDICINE *exhibited not a single instance of individual success or independance from such a source alone. When, therefore, the Editor can safely assert, that the* MEDICO-CHIRURGICAL REVIEW *returns him a nett revenue, at the present moment, of* ONE THOUSAND POUNDS PER ANNUM,* *he has just reason to be grateful to* PROVIDENCE *for sparing him health, and to the* PUBLIC *for awarding him so liberal a recompense for his labours. That these feelings of gratitude are mingled with some degree of pride, (not vanity) while taking a retrospect of the perilous undertaking which he instituted at his own risk, and compassed by his own exertions, must be candidly confessed—and will, he hopes, be pardoned, as one of those weaknesses from which human nature is seldom entirely free.*

---

* *The last quarterly sale, from the books of Messrs. Burgess and Hill, was* 1575, *amounting to the sum of* £340. 5*s. to which was added,* £26, 10*s. received for advertisements. The expence of the number was exactly* £100. *sterling. The balance may be easily calculated.*

*It has been recently stated, by an extra-professional cotemporary of some celebrity, that—" in periodical literature, there is no nonage, no lisping feeble immaturity. It springs at once to its full strength, like the rainbow. Its wisdom is, as it were, an intuition, and has no infancy. It changes like the sky; but it always preserves its due elevation." If our experience and observation have not completely deceived us, the above picture is false. Periodical literature or science is, like every other species of literature or science,—*improvable *by time, by habit, by practice, by discipline. By these, and these only, can we acquire a knowledge of our own powers, of the public taste, of the principles of our art, and a facility of composition. The history of periodical medicine shews us, also, many unequivocal instances of decrepitude and decay, from causes perfectly explicable upon the abovementioned principle.*

*A journal of medicine is generally allowed to be an engine of considerable power, and an institution of some consequence. It is not to be supposed, that it is exempt from the influence of agents which operate upon other human institutions and productions. Of the motives or springs which have, from time to time, set these machines in action, the most powerful and permanent are, we imagine, the love of science, the love of reputation, and the love of money. These, not even excepting the* last, *are perhaps the best springs or motives which can actuate such a machine. Unfortunately, a host of other motives and impulses have too often engrafted themselves on the original or legitimate ones, and contributed to the deterioration or even downfal of the machines themselves. It is impossible to take a survey of periodical medicine from the year* 1730, *when, we believe, it first commenced,—to examine the rise, progress, and declination of the numerous journals which, in* 90 *years, have figured on the literary stage—and not be convinced, that it was to good motives and honorable conduct, rather than splendid talents, they were indebted for their ascension—and that it was by corrupt principles or downright negligence, rather than defect of ability, they fell into oblivion. A careful examination of these records of periodical medicine, from the commencement of the physical and literary essays, down to the present time, (amounting to more than* 300 *volumes) has afforded us many instructive lessons, and delineated, as it were on a chart, the sources of their prosperity, and the rocks or quicksands on which they successively perished. We hope and trust, that we have profited by this examination, and that we shall*

*not turn to the right hand nor to the left, but steadily pursue the path inwhich zeal, industry, and good intentions, (to which we trust we may lay claim) have hitherto guided us in safety and with success.*

*On reviewing the three volumes now completed of this series, we have a firm conviction, that no equal number of volumes in the English language, contain a more concentrated mass of useful information for all classes of the profession; and, for the truth of this, we appeal to every unbiassed reader. We are also convinced that, should the work continue to be so conducted, and so encouraged, it is calculated to effect a great diffusion of medical and chirurgical knowledge through the middling and inferior ranks of our profession, where it is most needed. But to effect this very useful purpose, we must abandon all attempt to surprize our readers by novelty, or to give the earliest intimation of every thing engendered in the brains, or effected by the hands, of the myriads who are at work throughout the world. We must follow, not lead, the steps of science, and endeavour to keep up attention to what is true, rather than to what is new, in medical literature. Some years' experience has now proved that, by selecting a single department, (the analytical) and cultivating with assiduity the means of improving the said department, we shall effect more, than by aiming at a greater variety of entertainment for the public. And, under this conviction, we shall take leave of our readers for the present, promising that no exertions, on our parts, shall be spared in rendering the ensuing volume more valuable, if possible, than any of its predecessors.*

28*th February,* 1823.

